# The Rule of Law Conditionality Under Regulation No 2092/2020—Is it all About the Money?

**DOI:** 10.1007/s40803-021-00154-6

**Published:** 2021-04-13

**Authors:** Justyna Łacny

**Affiliations:** Department of Administration and Social Sciences, Warsaw University of Technology, Warsaw, Poland

**Keywords:** Conditionality, European values, Financial interests of the Union, Suspension of EU funds, Rule of law, Union budget, Union funds

## Abstract

Some say that the Union is built by moving from crisis to crisis. Crises in the last decade which affected the Union and its citizens concerned, *inter alia*, public finance (the financial crisis, 2008), migration (2014), public health (the COVID-19 pandemic, 2020) and the rule of law crisis (2018). This paper focus on the latter. It has been noted that some Member States have been happy to receive the benefits of EU membership, specifically the financial ones, while their commitment to European values, including the rule of law (Article 2 TEU), has been lacking. Since many instruments applied by EU institutions to improve this situation have proved rather insufficient, halting transfers of EU funds to these recalcitrant Member States has been touted as the way that might solve this crisis. Accordingly, a draft regulation was put on the table that authorised the EU institutions to suspend EU funds if a Member State is found to be in breach of the rule of law. This draft aimed to make the transfer of EU funds to the Member States conditional upon their continuous respect for the rule of law (and therefore became known as ‘the rule of law conditionality’). This paper comments on this draft as first proposed by the Commission in 2018 (Proposal for a regulation of the European Parliament and of the Council on the protection of the Union budget in the event of generalized gaps in the rule of law in the Member States [COM (2018) 324 final).], amended in 2019 by the European Parliament [European Parliament legislative resolution of 4 April 2019 on the proposal for a regulation of the European Parliament and of the Council on the protection of the Union's budget in case of generalised deficiencies as regards the rule of law in the Member States (COM(2018)0324–C8-0178/2018–2018/0136(COD)); https://www.europarl.europa.eu/RegData/seance_pleniere/textes_adoptes/provisoire/2019/04-04/0349/P8_TA-PROV(2019)0349_EN.pdf. A draft version of these provisions was presented in von Bogdandy and Łacny (Suspension of EU funds for breaching the rule of law - µ a dose of tough love needed? European Policy Analysis 2020, No 2, p. 1–15, https://sieps.se/en/publications/2020/suspension-of-eu-funds/, 2020).], and finally adopted by the European Parliament and the Council as Regulation (EU, Euratom) 2020/2092 of 16 December 2020 on a general regime of conditionality for the protection of the Union budget [Hungary and Poland voted against it and it is expected that its validity will be challenged before the CJEU via an action for annulment (Article 263 TFEU).] (henceforth called ‘Regulation 2020/2092′). This Regulation, containing 29 recitals in the preamble and 10 articles, entered into force on 1 January 2021 (Article 10 Regulation 2020/2092.). In the conclusions of the European Council meeting in December 2020 it was however accepted that it will be applied only in relation to budgetary commitments starting under the new Multiannual Financial Framework (MFF) 2021–2027, including Next Generation EU [Conclusions of the European Council meeting, 10 and 11 December 2020, para I (2) (k) https://www.consilium.europa.eu/media/47296/1011-12-20-euco-conclusions-en.pdf.]. This paper provides the legal characteristics of rule of law conditionality established under Regulation 2020/2092 and aims to determine whether financial incentives can restore compliance with the rule of law in Member States. Or in other words, is it all about the money?

## Introduction

The rule of law is the backbone of modern constitutional democracies and ensures that all public powers act within the constraints set out by law, in accordance with the values of democracy and fundamental rights and subject to the control of independent and impartial courts.^1^ This rule occupies a prominent place in EU; it is one of its core values.^2^ It has been stressed that the respect for the rule of law must be ensured throughout all EU policies, including EU finances,^3^ and this rule should be put at the heart of the EU budget.

In recent years however, some Member States have continuously violated the rule of law. It is noted that the Member States with a lengthy history of infringements of the rule of law receive extensive amounts of Union funds, which are significant drivers for their social and economic growth.^4^ For example, Poland is the largest overall recipient. In the MFF 2014–2020 Poland was allocated 86 billion EUR from various European Structural and Investment Funds (ESIF), and in the MFF 2021–2027 will receive €124 billion from the EU budget and up to €160 billion in loans.^5^ At the same time, Hungary is the largest recipient of Union funds on a *per capita* basis, with more than 95% of all public investments in the MFF 2014–2020 co-financed by the EU.^6^ EU funds transferred to Poland and Hungary were worth 3% and 4.55% respectively of those countries’ GDP in 2019. In the last decade, the populist parties governing in these States have increased state control over other public institutions, especially national prosecution and judiciary and the media. This situation has been dubbed as ‘rule of law backsliding’, meaning the process through which elected public authorities deliberately implement governmental blueprints which aim to systematically weaken, liquidate, or capture internal checks on power, with a view toward dismantling the liberal democratic state and entrenching the long-term rule of the dominant party.^7^ The Court of Justice of European Union (CJEU) has already found breaches of the rule of law in some cases brought against these States, all related to the independence of the judiciary.^8^ The CJEU has responded to the problem of restricting judicial independence by combining Article 2 TEU, as the legal basis for respect of the rule of law, with Article 19 TEU, stipulating that the principle of effective judicial protection gives concrete expression to the value of the rule of law.^9^ These victories, however, have not resulted in a return to the status *quo ante*, nor in restoring the institutional arrangements that were revised by the illiberal reforms. All the EU institutions have managed to achieve so far may be considered as tweaks, or cosmetic interventions removing the most offensive consequences of illiberal institutional reforms, while leaving the rest of the changes in place, essentially with the EU’s stamp of approval. ‘Creative compliance’ has been elevated to a new level.^10^ Oddly enough, voters in both countries remain strongly Europhile.

Hungary and Poland are not the only *enfants terribles* in the EU. Corruption allegations swirl in Bulgaria. High-profile murders of investigative journalists in Malta and Slovakia have shaken both countries lately. Croatian authorities are accused of beating up refugees at borders. EU leaders, however, are reluctant to interfere in their domestic affairs for the simple reason that they fear they could be next. In such circumstances, abstention becomes appealing.^11^

Against this backdrop, numerous measures and procedures have been developed over the years in the Union’s ‘rule of law toolbox’, aimed at safeguarding observance of the rule of law. They include*, inter alia*, the Article 7 TEU procedure designed to safeguard the Union’s founding values from serious and systemic breaches; the above-mentioned general infringement action brought to the CJEU to ensure the proper implementation of EU law (Article 258 TFEU); the EU Justice Scoreboard, which allows for rating the efficiency of national justice systems; and new annual ‘rule of law cycle’, with the first Commission’s report published in 2020, highlighting deficiencies in all Member States and leading to peer-to-peer dialogue in the Council, but with no follow-up measures provided for.^12^

The literature on measures and procedures available to EU institutions to safeguard the rule of law from violations by Member States is vast. There are a few basic points worth highlighting. First, the Union’s ‘rule of law toolbox’ is not the product of strategic engineering: it is a collection of several instruments developed over the years that may be used to safeguard the Union’s values. Many of these tools were not developed as specialized instruments to safeguard the rule of law as such but turned out to be helpful in practice in this respect (e.g. an infringement action). Without further refinements and adjustments, they cannot be expected to deliver their benefits—whether intended or ‘accidental’—consistently. Second, the Commission’s tools are complemented by the measures and procedures available to other EU institutions, sometimes independent of the Commission’s actions (e.g. the Parliament’s own initiatives) and at other times in response to the Commission’s actions (e.g. infringement actions). Third, the ‘rule of law toolbox’ and accompanying procedures have evolved over time, often against a backdrop of serious contestations regarding their legal basis as well as their appropriateness. The deep-seated belief that the EU institutions have moderate tools to sanction Member States for violating the rule of law, is hard to counter.^13^

In this narrative it is often claimed that it is ‘a curious omission’ that the Union does not insist more effectively on observance of the rule of law, e.g. as a condition for receiving Union funds. Some suggest that the suspension or cutting-off of Union funds would be a significant motivator for Member States to restore the rule of law, as well as a clear political message that the Union does not subsidize Member States that violate it.^14^ According to more radical voices, without the rule of law conditionality the EU funds not only support a cosy enclave of illiberal rulers: they enable them to consolidate their reach into the future, while side-lining the efforts of the counter-majoritarian (accountable) elements that are essential for constitutional democracy.^15^ Others, however, doubt that the external imposition of legal and political standards and linking them to financial sanctions can result in a profound transformation of legal culture and political behaviour unless accompanied by a real commitment and support in the respective Member State.^16^ This latter approach, however, did not prevail, and in response Regulation 2020/2092, which is examined in this paper, was adopted to allow the EU institutions to make transfers of Union funds to Member States conditional on their respect of the rule of law.

This paper concentrates on these above-mentioned issues. It introduces the concept of conditionality as applied in EU law so far (point 2). Then it presents its legal characteristics when applied to Union funds under Regulation 2020/2092. It discusses the definition of the rule of law and which types of breaches would launch the rule of law conditionality; the procedure and measures to be applied in such a situation; and the desired impact of these measures on the Member States; as well as the undesired, but possible, impact of these measures on end beneficiaries of Union funds (point 3).

## Conditionality in EU law

In the United States system conditionality has been effectively used since the 1940s to foster compliance on rule of law issues, in particular on issues such as corruption, to protect the independence of state or local civil servants managing federal funds, as well as to enforce the prohibition of segregation in educational establishments.^17^ In the EU, conditionality is a policy tool which has been applied only since the late 1980s.^18^ Its core tenet is that Member States are prompted to comply with the requirements established under EU law in return for advantages (including financial ones) to be received. Additionally, compliance should continue because, if it does not, the States risk losing their advantages (including the financial ones).

Conditionality was initially applied in the Union's external relations: the Union made the granting of humanitarian aid to third countries conditional upon their respect for human rights.^19^ Increasingly, conditionality is being used in the EU's internal relations as well. ‘Macroeconomic conditionality’ was introduced in 1994 to support the establishment of the European Monetary Union. Its conditioned access to the Cohesion Fund on compliance with the EU budgetary deficit rules. It has been enforced only once—against Hungary in 2012, when EUR 495 million from the Cohesion Fund was suspended for three months.^20^

In the MFF 2014–2020, two new types of financial conditionality were adopted: *ex-ante* conditionality and *ex-post* macroeconomic conditionality. To receive initial payment from the ESIF under ‘*ex-ante* conditionality’, the Member States had to fulfil specific conditions related to their capacity to properly absorb these funds by end of 2016. The EU funds could be suspended if it was discovered that the Member States had stopped fulfilling these conditions. According to ‘*ex-post* macroeconomic conditionality’, if Member States suffer from macroeconomic imbalances during the budget cycle, the Commission can ask them either to rearrange their plan for the ESIF or suspend it.^21^

The literature on conditionality suggests that financial assistance that is not backed by credible sanctions results in only formal compliance—often in a manner that undermines the overall aspirations of the assistance (e.g. building strong democratic institutions).^22^ Such use of conditionality in EU law marks a shift towards a more generalised ‘conditionality culture’ in the relations between the Union and its Member States. The compliance function that conditionality is aimed to ensure was initially intended to be fulfilled by the principle of sincere cooperation (Article 4 (3) TEU). The shift from the principle of sincere cooperation to that of conditionality was brought about by the 2004 enlargement, prompted by a concern that some ‘new’ Member States might be reluctant to fully fulfil their EU obligations.^23^ Calls for the establishment of rule of law conditionality, which allows for the suspension of Union funds granted to Member States that breach the rule of law, goes further down this route.

## The Rule of Law Conditionality Under Regulation 2020/2092

Regulation 2020/2092 establishes rules necessary for the protection of the Union budget in the event of breaches of the rule of law in Member States.^24^ This rule of law conditionality requires that breaches must affect or seriously risk affecting the sound financial management of the Union budget or the protection of the financial interests of the Union in a sufficiently direct way.^25^ The purpose of Regulation 2020/2092 is thus twofold and interconnected: to protect the Union budget, as well as to safeguard the rule of law in Member States. This limitation restricts the area of application of this Regulation only to situations wherein breaches of the rule of law by a Member State have a ‘sufficiently direct’ impact on the Union budget. The words in quotation marks did not feature in the Commission’s initial proposal. Linking the rule of law with the need to protect the Union’s financial interests—which manifestly limits the scope of rule of law conditionality—came about through the European Council’s conclusions in July 2020,^26^ at the beginning over the objections of Poland and Hungry.^27^

While Regulation 2020/2092 establishes a clear connection between rule of law violations and the financial interests of the Union, nevertheless its scope is still wide. The key consideration here is the degree of proximity required. The underlying idea is that there is a strong link between, on one hand, respect for the rule of law, and on the other hand mutual trust and financial solidarity among the EU and the Member States. This condition is therefore fulfilled when a violation of the rule of law is liable to directly affect that mutual trust. The impact on the EU interests must be direct, but not necessarily actual; a potential effect suffices. Political interference with the prosecution of crimes other than financial crimes may not meet the test of directness. However, if such interference is likely to create a climate of fear or occurs at a senior level of the prosecutorial or judicial authority, it may pass the threshold. The more fundamental or systemic the breach is, the easier it is to satisfy the requirement of directness.^28^

### Conditions for Applying the Rule of Law Conditionality

#### The Union Budget and the EU Recovery Instrument

As already noted, a need to protect the Union budget against damages resulting from breaches of the rule of law that affect or seriously risk affecting the EU budget is a necessary condition for the initiation of rule of law conditionality. According to Financial Regulation no 2018/1046^29^ (the main EU secondary budgetary law act), the Union budget is an annual financial plan of the EU and the European Atomic Energy Community, drawn up according to budgetary principles that provide forecasts and authorise, for each financial year, an estimate of future costs and revenue and expenditures and their detailed description and justification.^30^ The TFEU entrusts the task and responsibilities for implementation of the Union budget to the Commission.^31^ Implementation of the Union budget, or in other words its implementation via expenditure and revenue operations, requires the Commission to carry out different administrative actions related to the funds included in the Union budget e.g., to manage, monitor, control, and audit them.^32^

Beside the Union budgets (in MFF 2021–2027 €1074.3 billion), the rule of law conditionality also applies to resources allocated through the EU Recovery Instrument,^33^ established to counter the economic impact of the COVID-19 epidemic (Next Generation EU,^34^ €750 billion) and through loans and other instruments guaranteed by the Union budget.^35^

Regulation 2020/2092 highlights two notions related to Union funds: the principle of sound financial management,^36^ and the protection of the financial interests of the Union.^37^ The principle of sound financial management concerns the implementation of the Union budget. According to the TFEU, the Commission implements the Union budget in cooperation with the Member States, on its own responsibility and within the limits of the appropriations, showing proper regard for the principle of sound financial management.^38^ Financial Regulation no 2018/1046 further explains that according this principle, the Union budget must be implemented in accordance with the principles of economy, efficiency, and effectiveness.^39^ The CJEU has held that the principle of sound financial management (applied to the EU funds) corresponds to the principle of sincere cooperation (as applied more generally in EU law).^40^ The CJEU often recalls the prior principle in the context of EU rules that establish tasks related to the management of EU funds to underline the necessity of ensuring the legality of their collection and distribution.^41^ Regulation 2020/2092 establishing the rule of law conditionality develops the meaning of the principle of sound financial management. It states that it can only be ensured in Member States if public authorities act in accordance with the law, and if cases of fraud, including tax fraud, tax evasion, corruption, conflict of interest or other breaches of the law, are effectively pursued by the investigative and prosecutorial services, and if arbitrary or unlawful decisions of public authorities, including law-enforcement authorities, can be subject to effective judicial review by independent courts and by the CJEU.^42^ Regulation 2020/2092 thus links the principle of sound financial management not only to the legality of administrative operations, but also to judicial independence as well as to effective prosecutions.

The core notion of ‘protection of the financial interests of the Union’ comes from the TFEU,^43^ and has been defined in EU secondary law^44^ and by the CJEU. The financial interests of the Union cover all revenues, expenditures and assets included in the Union budgets. These financial interests include all the budgets of the EU institutions, bodies, offices and agencies established under the EU Treaties. Article 325 TFEU—the main Treaty provision relating to the financial interests of the Union—obliges the Member States and the EU institutions to counter fraud and any other illegal activities affecting these interests through deterrence measures; and that such measures should ensure the effective protection of the financial interests of the Union. In turn, the Member States should take measures to counter fraud that affects the financial interests of the Union in the same way as they counter fraud affecting their own financial interests (the assimilation principle).

Regulation 2020/2092 provides further clarifications concerning the obligations to protect financial interests of the Union. Namely, it determines minimum guarantees against unlawful and arbitrary decisions of public authorities that could harm these interests. These guarantees include providing the judiciary, investigative and prosecutorial services with sufficient financial and human resources and procedures to act effectively and in a manner that fully respects the right to a fair trial, as well as effective implementation of the final judgments.^45^ It is no accident that the Regulation 2020/2092 emphasizes the effectiveness of the judiciary as well as the investigative and prosecutorial services.

#### The Rule of Law and its Breaches

A breach of the rule of law by a Member State is a second condition—next to the need to protect of the Union budget against such a breach—necessary to apply the rule of law conditionality under Regulation 2020/2092. The ‘rule of law’ is defined, for the purpose of this Regulation, as relating to the Union values and principles enshrined in Article 2 TEU. The rule of law implies legality and a transparent, accountable, democratic and pluralistic law-making process; legal certainty; the prohibition of arbitrariness by the executive powers; effective judicial protection, including access to justice, by independent and impartial courts (also as regards fundamental rights); separation of powers; and non-discrimination and equality before the law.^46^ Regulation 2020/2092 makes clear that the notion ‘the rule of law’ must be understood as it is commonly accepted in the jurisprudence of the CJEU, by including series of references to the relevant case-law. Through this Regulation, the Council, i.e. all Member States, strongly support the path-breaking case-law the CJEU has developed over the last years.

The preamble of the Regulation 2020/2092 explains that while there is no hierarchy among the Union’s fundamental values (Article 2 TEU), respect for the rule of law is essential for the protection of the other values on which the Union is founded, such as freedom, democracy, equality and respect for human rights. It states that respect for the rule of law is intrinsically linked to respect for democracy and to the protection of fundamental rights. There can be no democracy and respect for fundamental rights without respect for the rule of law, and vice versa.^47^

Regulation 2020/2092 provides an open list of examples indicative of a relevant breach. These examples include endangering the independence of the judiciary; failing to prevent, correct or sanction arbitrary or unlawful decisions by public authorities, including by law-enforcement authorities; withholding financial and human resources affecting their proper functioning or failing to ensure the absence of conflicts of interest; and limiting the availability and effectiveness of legal remedies, including through restrictive procedural rules and lack of implementation of judgments or limiting the effective investigation, prosecution or sanctioning of breaches of law.^48^ It continues with an indication of the area(s) of the Member States’ functioning that may be affected by a breach of the rule of law. These areas include, e.g., the proper functioning of the authorities implementing the Union budget; the proper functioning of the authorities carrying out financial control, financial management, monitoring and auditing; the proper functioning of investigative and public prosecutions in relation to fraud, including tax fraud, corruption or other breaches of Union law relating to the implementation of the Union budget or to the protection of the financial interests of the Union; and finally effective judicial review by independent courts of actions or omissions by the authorities referred to above.^49^

It can be seen at first glance that many examples of the listed breaches of the rule of law by the Member States relate to the effectiveness of the national prosecutorial services and the independence of judiciary. Regulation 2020/2092 clearly stipulates that Article 19 TEU, which gives concrete expression to the value of the rule of law (Article 2 TEU) requires the Member States to provide effective judicial protection in the fields covered by Union law. It further explains that the very existence of effective judicial review designed to ensure compliance with Union law is the essence of the rule of law and requires independent courts.^50^ Such independence of the judiciary presupposes, in particular, that the judicial body concerned is able to exercise, both under the relevant rules and in practice, its judicial functions wholly autonomously, without being subject to any hierarchical constraints or subordinated to any other body, and without taking orders or instructions from any source, thus being protected against external interventions or pressures liable to impair the independent judgment of its members and/or to influence their decisions. These guarantees of independence and impartiality require rules—particularly with respect to the composition of the relevant bodies and the appointment, length of service, and the grounds for rejection and dismissal of its members—in order to dismiss any reasonable doubt in the minds of individuals as to the imperviousness of that body to external factors and ensure its neutrality with respect to the matters before it.^51^ The influence and inspiration of these provisions in the jurisprudence of the CJEU is clearly visible in the cases concerning the breaches of the rule of law by the Hungary and Poland, as well as in references to the problematic situations of the prosecutorial services and judiciary in both these states.

In comparison to the draft Regulation,^52^ Regulation 2020/2092 gained in clarity but limited the material scope of the rule of law conditionality to breaches related to EU funds only. At the same time, Regulation 2020/2092 drops the requirement that the rule of law violation must be systemic or repetitive. As the preamble makes clear, a relevant rule of law breach can be found in an individual act.^53^ It thus appears that even an individual violation of the rule of law suffices to apply the rule of law conditionality. In comparison, the draft Regulation required that the rule of law conditionality could be initiated only if ‘generalised deficiencies as regards the rule of law’ were found in the Member States. Such a ‘generalised deficiency’ was defined as a systematic conduct of public authorities in a Member State in violation of the rule of law which affects or is likely to affect EU funds or their management. Such violations must have had a systematic or a common character in a Member State.^54^

### Legal Basis

The Regulation 2020/2092 establishing the rule of law conditionality is based on Article 322 (1) (a) TFEU. This article, included in the part of this Treaty containing financial provisions, authorizes the European Parliament and the Council to adopt regulations or procedures for establishing and implementing the Union budget. The objective of the rule of law conditionality is to protect of the Union budget against the harm—possible or real—resulting from rule of law breaches by the Member States. The selection of Article 322 (1) (a) TFEU for the legal basis of the Regulation 2020/2092 may indicate that protection of the Union budget is the primary objective of this Regulation, while the breaches of the rule of law indicate the scale of the protection to be ensured.^55^ This reasoning relates to the scope of application of Regulation 2020/2092, which must be connected ‘in a sufficiently direct way’ with damage caused or to be caused to the Union funds by the breach of the rule of law. This conclusion can also be confirmed by the institutional framework proposed therein, in which the Commissions plays an important role. By checking the rule of law observance by the Member States and proposing to launch the rule of law conditionality if breaches are detected, the Commission would perform its dual role as both the EU institution responsible for implementation of the Union budget^56^ as well as the guardian of the EU Treaties.^57^

What can raise questions are the reasons why Article 325 TFEU—the main TFEU provision aimed to protect the financial interests of the Union—is not invoked as the legal basis of Regulation 2020/2092. Since both Article 322 (1) (a) TFEU and Article 325 (4) TFEU allow the European Parliament and the Council to adopt an act in accordance with the ordinary legislative procedure, after consulting the Court of Auditors, the reason may be the material scope of these two Articles. Since Article 322 (1) (a) TFEU applies to all aspects related to the implementation of the EU budget, it is wider than the scope of Article 325 TFEU, which relates only to protection of the budget.

### The Measures Under Rule of Law Conditionality

#### Types of Measures

Regulation 2020/2092 provides for several measures. They depend on the methods by which the Commission implements the Union budget, i.e. it either does so itself (directly or indirectly^58^), or in cooperation with the Member States (under shared management^59^). Where the Commission implements the Union budget (directly or indirectly) and a government entity^60^ is the recipient of the Union funds, the following measures can be applied to the Member State concerned: suspension of payments or of the implementation of the legal commitment, or termination of the legal commitment;^61^ a prohibition on entering into new legal commitments; a suspension of the disbursement of instalments in full or in part or an early repayment of loans guaranteed by the Union budget; a suspension or reduction of the economic advantage under an instrument guaranteed by the Union budget; or a prohibition on entering into new agreements on loans or other instruments guaranteed by the Union budget.^62^ Where the Commission implements the Union budget in cooperation with the Member States (under shared management), the following measures can be imposed on the Member State concerned: suspension of the approval of one or more programmes or an amendment thereof; a suspension of commitments; a reduction of commitments, including through financial corrections or transfers to other spending programmes; a reduction of pre-financing; an interruption of payment deadlines; or a suspension of payments.^63^ Regulation 2020/2092 makes clear that one or more such measures can be imposed on the Member States breaching the rule of law.^64^

The above measures can be taken at various stages of the implementation of EU policies: from the stage of approval of national programmes by the Commission to the stage of making legal commitments by the Commission, followed by the payments of the EU funds to the Member States, and to their recovery if irregular expenditures are detected. However, Regulation 2020/2092 does not establish the characteristics of the measures mentioned nor stipulate how they should operate in practice. This requires a reference to the specific provisions included in sector-specific regulations providing rules for the expenditure of the EU funds for specific EU policies, e.g. the cohesion policy or CAP, which altogether consume circa 70% of the Union budgetary expenditures.^65^ To give just one example—one of the measures that can be imposed for breaches of the rule of law by a Member State is a reduction of commitments taken towards this State through financial corrections. If one would wish to ascertain what these financial corrections are and how they legally operate,^66^ there is no choice but to refer to the provisions of the cohesion policy and the CAP regulations that regulate them.

#### Selection of Measures

As mentioned above, the essence of the measures to be adopted and applied to the Member State breaching the rule of law can be specified by referring to sector-specific regulations (see point: 3.6). Regulation 2020/2092, unlike the previous draft, clearly refers to these legal acts, which require approval. It states that the sector-specific and financial rules provide for various possibilities to protect the Union budget, including interruptions, suspensions, or financial corrections linked to irregularities or serious deficiencies in management and control systems. Importantly, it establishes the relationship between the application of sector-specific regulations and the rule of law conditionality. It states that Regulation 2020/2092 should be applied only in cases where the other procedures set out in Union law would not allow the Union budget to be protected more effectively.^67^ It follows that the rule of law conditionality established by Regulation 2020/2092 has a complementary function and can only be initiated if other instruments established under the Union law do not allow for the effective protection of the Union fund harmed by the breach of the rule of law. If it is decided that measures be imposed on the Member State under rule of law conditionality, their content would then be decided by the sector-specific regulations that regulate them. What is, however, regrettable is that despite the fact that these sector-specific regulations should come into force from 1 January 2021 (to apply to the MFF 2021–2027),^68^ their final shape of is so far unknown as they are still in legislative processes.

#### Criteria for Applying Measures

Regulation 2020/2092 provides the criteria which the EU institutions should consider when imposing measures on those Member States breaching the rule of law.^69^ These measures should be proportionate^70^ (adequate^71^), in particular with regard to the seriousness of the situation related to the detected breach of the rule of law, the time which has elapsed since the relevant conduct of the Member State started, the duration and recurrence of this conduct, as well as the intention and the degree of cooperation of the Member State in putting an end to the breach. Measures imposed should, insofar as possible, target the Union actions affected by the breach(es).^72^ It follows that the criteria for applying the measures under rule of law conditionality relate to both the breach of the rule of law (its seriousness, duration, recurrence) and the Member State’s approach to this breach (degree of cooperation and intentions).

In December 2020, to dilute the Polish and Hungarian opposition to the adoption of Regulation 2020/2092, the European Council adopted an ‘interpretative declaration on the new rule of law mechanism’.^73^ In this declaration it stated that with a view to ensure that the rule of law conditionality is applied objectively, fairly, and with due regard to the equal treatment of Member States, the Commission would develop and adopt—in close consultation with the Member States—guidelines on the way it will apply the Regulation 2020/2092, including a methodology for carrying out its assessment. It also stated that until such guidelines are finalized, the Commission will not propose measures under this Regulation. As regards the question of when these guidelines may be deemed to be finalized should an action for annulment be filed with the CJEU regarding Regulation 2020/2021 (as announced by Poland and Hungary), the declaration stated the guidelines will be finalized after the judgment of the CJEU. Despite the fact that this declaration was handed down in the European Council’s conclusions and is therefore is of a merely political character, some legal scholars claim that it has a legally binding force, that and by adopting it the European Council acted *ultra vires*.^74^

### Procedure

Regulation 2020/2092 provides for an adversarial procedure, in which a legal dispute concerning alleged breaches of the rule of law by the Member States is conducted between the Commission and the Member State concerned, resolved by the Council, and can subject to judicial assessment by the CJEU.

#### Who Takes a Decision?

Regulation 2020/2092 stipulates that the identification of breaches of the rule of law by a Member State requires a thorough qualitative assessment by the Commission. This assessment must be objective, impartial, and fair, conducted with respect to the principles of non-discrimination and equal treatment of Member States, and based on a non-partisan and evidence-based approach.^75^ The Commission must consider all relevant information from all available sources and recognized institutions.^76^

During the legislative works on Regulation 2020/2092 the European Parliament had proposed the establishment of an advisory panel of independent experts (the panel) that would assist the Commission with the assessment of the rule of law situations in the Member State(s).^77^ The panel was to perform two tasks. First, it would adopt ad hoc opinions on specific cases of rule of law breaches by a Member State brought to its attention by the Commission. Secondly, it would annually assess the health of the rule of law in all Member States and publish its findings. This proposal, however, was not included in Regulation 2020/2092.

The Regulation does however foresee that after the Commission conducts its assessment, and where it finds reasonable grounds for considering that the conditions for initiating the rule of law conditionality are fulfilled (see: point 3.1), it will send a written notification to the Member State concerned. In this notification the Commission will indicate the factual elements and the specific grounds on which it based its findings. According to the*audi alteram partem rule* the Commission may request any additional information from the Member State concerned to verify its findings, both before and after having sent a written notification.^78^ Then the Member State may make observations on the findings set out by the Commission and propose the adoption of remedial measures to address these findings.^79^ If, despite these clarifications, the Commission still intends to proceed, it will submit to the Council a proposal for an implementing decision on the appropriate measures to be imposed on the Member State (see point 3.3). This proposal should set out the specific grounds and evidence on which the Commission based its findings.^80^ Then the final decision is taken by the Council. The European Parliament, the second co-legislator, is informed about any Commission proposal submitted to the Council and a decision taken by the Council. It may invite the Commission for a structured dialogue on its findings.^81^

#### Majority in the Council

If the required conditions are met, measures are imposed on the Member States (and lifted if appropriate) in the form of an implementing decision.^82^ The Council adopts this decision on the request of the Commission by qualified majority vote (QMV),^83^ which is the standard voting procedure in the Council.^84^ This is a significant novelty in comparison to prior versions of these provisions. Originally, it had been foreseen that the Council would adopt these decisions by *reverse* QMV,^85^ which has no explicit basis in the EU Treaties. In the case of *reverse* QMV, a proposal submitted (by the Commission) is adopted unless the legislature (the Council) rejects or amends it by QMV in a vote held within a certain period. Following this period, a decision is considered adopted. That would mean that the Commission proposal would be deemed to have been adopted *unless* the Council *rejects* it by a QMV (within one month of its submission). The Council might also amend the Commission proposal by a QMV (with no time-limit established for this). Thus, in the case of *reverse* QMV, a failure to obtain the QMV necessary to block or change a proposal within a specific period results in its adoption (in the wording of the draft provisions, ‘the decision shall be deemed to have been adopted’). Hence under *reverse* QMV, abstention counts as a positive vote, forcing the Member States to take a clear position and obliging them to explicitly vote against the proposal, rather than following the more politically expedient route of abstention. It follows that if the Commission would seek to initiate the rule of law conditionality, it would be much easier to do so under *reverse* QMV then under QMV. According to prior explanations, the idea of voting on launching the rule of law conditionality by *reverse* QMV was dictated by the necessity to protect EU funds.^86^ It was also argued that *reverse* QMV was applied to avoid the unanimity requirement of Article 7 (2) TEU as well as the 4/5 majority required by Article 7 (1) TEU, both of which have been exploited by Hungary and Poland, who have formed a coalition in which each State made the imposition of sanctions against the other impossible.

However, had*reverse* QMV been adopted it would not have been the first time to have such voting in the Council as a remedy to ensure the effective implementation of Union law. Such voting was established in 2011 to strengthen the enforcement of the EU fiscal rules.^87^ Under these rules the Member States must avoid excessive government deficits (3% of GDP) and debt (60% of GDP) and maintain sound and sustainable public finances. In 1997 the Member States signed the Stability and Growth Pact (SGP) to facilitate the implementation of these fiscal rules by establishing a system to monitor their budgetary situation.^88^ In 2011, in the face of the world financial crisis, enforcement of the SGP was strengthened by the adoption of the ‘Six Pack’, ‘Two Pack’ and ‘Fiscal Compact’. These acts provided for financial sanctions: interest-bearing deposits, non-interest-bearing deposits, and fines to be imposed on the Member States for recurrent breaches of the EU fiscal rules.^89^ Under the Fiscal Compact, these sanctions are imposed by the Council by *reverse* QMV. Since the legality of the sanctions adopted for breaches of the EU fiscal rules under *reverse* QMV was not contested before the CJEU, its position on this issue is unknown. The inclusion in Regulation 2020/2092 of the rule that the Council adopts its decision by QMV (i.e. abandonment of the idea of voting by the effective but legally questionable procedure of *reverse* QMV) should mute the discussion on the illegality of this part of the act.

#### Lifting the Measures

The Member State on which the Council imposes a measure(s) under Regulation 2020/2092 (see point 3.3) may at any time present to the Commission remedial measures adopted to rectify the rule of law breach(es) and submit to it a written notification including evidence that the conditions for applying these measures are no longer met.^90^ The Commission reassesses the situation in the Member State concerned at the request of this State (at any time) or on its own initiative (up to 1 year after adoption of the measure(s), at the latest).^91^

The procedure is completed depending on the situation concerning the rule of law breach(es) which gave rise to it. If the Commission finds that conditions for imposing measures on the Member State are no longer met, it submits to the Council a proposal for an implementing decision lifting these measures. If the Commission determines that the situation leading to adoption of these measures has been remedied only in part, it proposes to the Council an implementing decision amending these measures. However, if the Commissions concludes that the situation leading to the adoption of the measures has not been remedied by the Member State, it addresses to this State a reasoned decision, including evidence supporting its findings. The Commission should provide this reasoned decision to the Member State within one month from receipt of a written notification from this State, or in duly justified circumstances within a longer period.^92^ Actions aimed at lifting, amending, or maintaining measures imposed on the Member State are carried out according the procedural requirements set out in Regulation 2020/2092 for their adoption.^93^

#### Time-Limits

Regulation 2020/2092 contains many time-limits for conducting specific procedural steps which, as one may assume, should speed up its completion and thus promote its effectiveness. At the beginning of the procedure—when the Commission requests the Member State to provide it with relevant information and observations on the findings set out in the written notification—the State concerned should deliver them within a period of one up to three months from the date of the notification.^94^ The Commission then has a one month from the receipt of this information to assess whether the conditions for the adoption of measures under the rule of law conditionality are met. If the Member States does not provide the Commission with the requested information, the Commission should make its assessment within a maximum of three months from the date of written notification, and in any event within a reasonable time frame.^95^ As a general rule, when the Commission sets up a time-limit for a Member State for the completion of procedural requirements, it should take into consideration the amount of information requested, the complexity of the relevant facts and their assessment, and the administrative capacity of the Member State concerned.^96^

Before the Commission proposes to the Council the adoption of an implementing decision imposing measures on a Member State, it should thus offer this State the opportunity to submit its observations within one month, in particular on the proportionality of the envisaged measures.^97^ If, despite the information obtained from such Member State, the Commission still finds that there are grounds for adopting such a decision, it submits its proposal to the Council. The Council should then adopt (or reject) this decision within one-month, which period of time may exceptionally be extended by a maximum of two additional months. To ensure that the Council takes its decision within these time-limits, the Commission may,^98^ if appropriate, convene a Council meeting and make the appropriate use of its rights under the Council’s Rules of Procedure.^99^

As already stated, the procedure for adopting (or lifting) a decision imposing measures on the Member States should be conducted in accordance to the principle of objectivity, non-discrimination, and equal treatment of Member States, and be based on a non-partisan and evidence-based approach. If the Member State considers that any of these principles were seriously reached, it may request the President of the European Council to refer the matter to the next European Council meeting. In such circumstances, no decision is taken until the European Council has discussed the matter. This should, as a rule, take no longer than three months after the Commission has submitted its proposal to the Council.^100^ One may ask how many times a Member State may exercise this right – only once or repeatedly within the same procedure? The need for a prompt conclusion to the proceedings, reflected in the establishment of the above time-limits, would lead to the conclusion that that this right should be a one-off.

### Impacts

#### Impact on the Member States

The impact of the measures imposed on the Member States under the rule of law conditionality depends on the type(s) of the measure(s) adopted. As already stated, determination of the essence of these measures, including the possible impact they may have, requires reference to the sector-specific regulations that regulate them (see point 3.6). Regulation 2020/2092 explicitly provides for the impact of two measures imposed if EU funds are spent under shared management, namely the suspension of the approval of programmes or amendments thereof,^101^ and the suspension of commitments.^102^

The first measure requires the approval by the Commission of (operational) programmes presented to it by the Member States or amendments of the programs already approved. A requirement to obtain such approval(s) applies within the cohesion policy and in the CAP (second pillar). This approval is required to start spending (in the case of approval of programs at the beginning of the MFF) or to continue spending (in case of approval of amendments of programs already approved) the Union’s structural funds. Suspension of approvals would mean that the Commission will not be authorised to start the transfer or continue making transfers of EU structural funds to a Member State. The second measure is a suspension of the (budgetary) commitments. A budgetary commitment is a reservation of budgetary appropriations (payments) to cover subsequent expenses from the budget. If such commitments are not reserved, there will be no financial resource available to cover the budgetary appropriations.^103^

When it comes to the impact of these two measures on the Member States, according to Regulation 2020/2092 if they are lifted the amount of Union funds corresponding to the suspended commitments are entered in the Union budget. In other words, they are returned to the Union budget that they came from. But what is crucial in this respect is that commitments suspended within a year ‘n’ may not be entered into the Union budget beyond year ‘n’ + 2.^104^ Thus, in principle, suspension of commitments should only lead to a temporary suspension of Union funds, thus motivating errant Member States to promptly eliminate the breaches of the rule of law giving rise to the suspension. If the situation improves, the commitments suspended in a certain year (‘n’) by the Council may be re-committed by the Council and then re-entered in the Union budget by the Commission within two following years (year ‘n’ + 2). Thus, the amounts of Union funds corresponding to the suspended commitments are returned to the Member State and may be reutilized by it. However, after lapse of these 2 years the Commission will no longer have a legal basis to enter these amounts into the Union budget as earmarked for the Member State concerned in the MFF. These funds would be then available to all Member States. It thus follows that a Member State whose commitments to be paid by Union funds have been suspended has only 2 years from the year of suspension (year ‘n’) to remedy the rule of law breach(es). If the Member State fails to undertake the required actions during this 2-year period, it loses the suspended Union funds. Conducting all these actions within 2 years could be difficult, taking into consideration that the elimination of breaches of the rule of law, particularly if a breach is a long-lasting and systemic one, is usually time-consuming, and in addition further time is needed for the proceedings in the Council and the Commission to lift the suspension. It follows that in these cases the suspension of EU funds could relatively easily result in their permanent loss.^105^

The impact of other measures that can be imposed on a Member State breaching the rule of law requires recourse to the sector-specific regulations that regulate these measures. As already mentioned, these regulations are still in the Union legislative process. It can however be stated that the very names of these measures suggest that they will be able to lead to a suspension of the Union funds as well as to a definitive end of Union payments and their recovery from the Member States. If Union funds are spent under direct management, such an impact could be achieved*prima facie* in cases of: termination of the legal commitment^106^; prohibition of entering into new legal commitments^107^; reduction of the economic advantage under an instrument guaranteed by the Union budget^108^; and a prohibition against entering into new agreements on loans or other instruments guaranteed by the Union budget.^109^ In cases of spending Union funds under shared management, the same impact can be achieved in cases of reduction of commitments, including through financial corrections.^110^ The legal nature of these measures has not yet received an in-depth analysis by the CJEU or legal scholars. However, it can be said that the essence of these measures, especially the reduction of the commitments, goes far beyond a mere suspension of the payment of EU funds and implies their irretrievable loss. Therefore, the rule of law conditionality suspension established under Regulation 2020/2092 could, contrary to its name, lead not only to the suspension of EU funds, but also to their definitive loss by a Member State violating the rule of law.

#### Impact on the Beneficiaries

In principle, the measures imposed under rule of law conditionality (see point 3.3.) should solely affect the Member State breaching the rule of law. These measures should not in any way influence the end-beneficiaries of these funds (e.g. local governments, entrepreneurs, employees, farmers). During the legislative works on Regulation 2020/2092 it was however pointed out that the suspension or withdrawal of EU funds to the Member States breaching the rule of law, or to be more specific of funds to their governments responsible for these violations, could easily end up penalizing the beneficiaries rather than these States, and thus in the end harm the very people the EU is trying to help or protect. This created a combined practical and legal question about how to prompt the government to improve the rule of law situation without involuntarily punishing its citizens.

To remedy this situation, the prior version of what is now Regulation 2020/2092 foresaw that, unless the decision adopting measures on the Member State provides otherwise, the imposition of these measures should not affect the obligation of the Member State concerned to implement the programmes or funds affected by the measures and, in particular, the obligation such a State has towards beneficiaries, including to make payments to them. This was intended to ensure that if measures are imposed, the Member State must from its own revenue finance the costs of implementing the Union programmes financed until then by Union funds and make payments to beneficiaries. However, the lack of rules giving effect to the protection of beneficiaries against the possibility that the Member State would cease to make payments to them was often criticized.^111^

To strengthen the legal protection of beneficiaries, the European Parliament^112^ proposed that the Commission should provide guidance for them, via a website, on the obligations of Member States to implement the Union programmes and make payments to them. This website should provide beneficiaries with tools to inform the Commission of any breaches by a Member State of its obligations. Furthermore, beneficiaries were to be protected under the whistle-blower rules.^113^

These and further rules were included in Regulation 2020/2092 to ensure proper safeguards of the legitimate interests of beneficiaries if Union funds are spent under shared management. It can be recalled here that in the case of shared management, payments from the Commission to Member States are legally independent from payments by national authorities to beneficiaries. In contrast, if Union funds are spent under direct management, the Commission itself makes the payments to beneficiaries and undertakes other management tasks (selecting contractors, awarding grants, transferring funds, etc.).^114^ Regulation 2020/2092 clearly stipulates that in the case of shared management, the Member State must implement the programmes or Union fund affected by the measures imposed and fulfil the obligations they have towards beneficiaries, including making payments required under the Regulation and sector-specific rules. In addition, the Member State must also report to the Commission on its compliance with above obligations every three months after the Commission’s adoption of measures. From its side, the Commission should provide information and guidance to the beneficiaries on the obligations by Member States via a website. Adequate tools should be set up on this website to enable the beneficiaries to inform the Commission about any breach of obligations by a Member State which directly affects them.^115^ What’s important, and what is a real novelty, is that if the Commission finds the Member State has stopped making payments to beneficiaries, it may take appropriate measures towards this State in line with sector-specific rules,^116^ e.g. recover payments made or make a financial correction by reducing Union support to programmes in line.^117^ Violation of the interests of beneficiaries by a Member State may therefore lead to a reaction from the Commission and may result in further suspensions or cut-offs of EU funds.

### Sector-specific regulations on the cohesion policy and the CAP

Long before adopting Regulation 2020/2092 allowing for the suspension of Union funds transferred to the Member States, it was pointed out that EU secondary law already provided for such a possibility.^118^ However, while it was rather clear that the Commission had such competences in external relations, i.e. when a third country breached the rule of law,^119^ it was disputable whether the Commission had such rights in internal relations if a Member State breached the rule of law. Some legal scholars claimed that such possibilities were indeed offered under the sector-specific regulations. They indicated that relevant provisions were included in the Common Provisions Regulation (CP Regulation)^120^ containing rules on the ESIF expenditures used to finance implementation of the cohesion policy. Comparable rules were also included in the CAP Regulation.^121^ Under both Regulations, the Member States receiving Union funds were obliged to establish a so-called ‘management and control system’ for spending these funds and ensure the effective functioning of these systems.^122^ Accordingly, these systems—comprised of national bodies undertaking specific tasks related to the distribution of EU funds in the Member State—should guarantee that the funds are spent in accordance with EU law. When the Commission detects weaknesses in the effective operation of the management and control systems which lead to irregular spending, the CP/CAP Regulations authorize it to conduct certain measures. If there is evidence to suggest a serious deficiency in the management and control systems, about which the Member State has not taken corrective measures, the Commission may interrupt the payments deadline.^123^ If there was a serious deficiency in the effective functioning of this system, the Commission may suspend the payment of Union funds to the Member State.^124^ Thus, the impact of both instruments, i.e. the CP/CAP Regulations and Regulation 2020/2092, is similar. In both cases Union funds are not transferred to the Member State until the situation improves. And finally, if there is a serious deficiency in the effective functioning of the management and control system, one which has put at risk the support from the EU funds already paid, the Commission may impose financial corrections on the Member State, leading to a reduction of EU funds to this State.^125^ It has been claimed that cases of undermining the judicial independence in Member States could be classified as a serious deficiency in the effective functioning of the management and control system. If so, such instances could lead to an interruption of the payment’s deadline, suspension of payments, and in the most serious cases to the recovery of EU funds already paid. The Commission however appears to have not explored the above possibilities provided under the CP/CAP Regulations. Instead, it has proposed the adoption of is now the Regulation 2020/2092 establishing the rule of law conditionality mechanism discussed in this paper.

Legal scholars also claim that the new CP/CAP Regulations for the MFF 2021–2027 should explicitly demand respect for the rule of law as a precondition for the receipt of EU funds, and provide for a suspension of EU funds if this rule is breached.^126^ It was similarly stated that access to EU funds should be limited only to those Member States that participate in the European Public Prosecution Office.^127^ However, none of these demands have been inserted into an EU legal act.

In conclusion it may be stated that the final shape of the sector-specific Regulations may influence the way the rule of law conditionality would be applied. For example, according to the latest draft CP Regulation, the Commission would be authorised to suspend payments made to the Member State in the event there is a reasoned opinion adopted under the general infringement procedure concerning EU law violations by the Member State that put at risk the legality and regularity of Union expenditures.^128^ This premise would create a direct connection between the general infringement action also used for protecting the rule of law (Article 258 TFEU) and access to EU funds, which eventually may also be suspended under the rule of law conditionality (Regulation 2020/2091). It follows that the Commission will have to analyse all the possible combinations of these procedures (i.e. Article 258 TFEU; rule of law conditionality; and sector specific Regulations) to decide which one would allow for the most effective protection of the Union's financial interests against the real or potential damage resulting from breaches of the rule of law by a Member State.

## Conclusions

Depending on one’s attitude, one can see the glass as half empty or half full. In the latter perspective, some positive aspects of the rule of law conditionality established under Regulation 2020/2092 can be observed. The most important point is that—following a heated political and legal debate which lasted 2 years—it was finally adopted and is now in force. Rule of law conditionality has been set up as a legal mechanism allowing for the protection of the Union budget in the case of breaches of the rule of law by Member States. This restricted area of application, often criticized as severely disappointing, may to a certain extent silence those who seen the glass as half empty, who point out that rule of law conditionality is another rule of law instrument with a vague scope of application and unclear relationship to the other instruments intended to serve this purpose. However there is a perception is that the final provisions contained in Regulation 2020/2092 have been strengthened compared to the original draft, which required the existence of a ‘general deficiency as regards the rule of law’ to launch the rule of law conditionality, while under Regulation 2020/2092 this seems possible even where a single breach of the rule of law is found to have occurred. Accepting Article 322 (1) (a) TFEU as an appropriate legal basis for the establishment of the rule of law conditionality – as its primary aim is to protect the EU budget against financial damages resulting from violations of the rule of law – may calm the discussion over whether it constitutes an legally unacceptable alternative measure to the Article 7 TEU procedure. It is not. The establishment in Regulation 2020/2092 of specific criteria directing the imposition of measures on the Member States breaching the rule of law, to which the principle of proportionality applies and is as well subject to judicial review by the CJEU, undermines arguments that this conditionality will be entirely at the discretionary power of the Commission or the Council. And the adoption of the rule that the Council would take decisions imposing measures on the Member States by QMV – and not the attractive but legally questionable *reverse* QMV – can be seen as having restored the proper order of things. The establishment of a time-limits for taking procedural steps allows one to have a silent hope that implementation of the rule of law conditionality will not be a ‘never ending story’ like the Article 7 TEU procedure. Measures imposed on Member States under the rule of law conditionality can certainly put financial pressure on them. This will depend on the seriousness of the breach of the rule of law and the decision of the Council as to whether these measure(s) would lead only to the suspension of EU funds or end-up in the much more severe obligation to repay Union funds already received by the Member States. Contrary to its name, due to the ‘n + 2′ principle, the rule of law conditionality may lead to suspension of EU funds, but also – which is surprisingly not widely discussed in the public debate – to their definitive loss by a Member State violating the rule of law. The legal situation of the beneficiaries of the Union funds living in a Member State against whom the rule of law conditionality would be applied has been improved. This is primarily because an enforcement measure has been added to the obligation of a Member States to disburse the EU funds to them, even if under the rule of law conditionality these funds were suspended, or in the most serious cases would have to be returned by the State concerned. On top of this the State must regularly report to the Commission on how it fulfils this obligation. Independently, the Commission will inform beneficiaries that the Member States may not stop making payments to them. And if so, adequate tools should allow the beneficiaries to inform the Commission about any such breach that directly affects them. What is crucial is that if the Commission finds the Member State to have really stopped making payments to beneficiaries, it would be authorised to take appropriate measures towards such State in line with sector-specific rules, e.g. recover Union payments already made or reduce Union support.

All these events may happen, but they don’t have to. The risk is that the rule of law conditionality becomes another Stability and Growth Pact – a bloc of mostly-ignored rules. Even in good times the SGP requirements were sometime not complied with, and the consequences were few. The Member States could be fined, but none ever was.^129^ In her song Meja asks if it's all about money? If so—she says—it means that we got it all wrong anyway. We probably got it all wrong in the first place if, in our ‘community of values’, we have to employ financial pressure to try to make misbehaving Member States respect them. We got it even more wrong if our realism causes us wonder whether the rule of law conditionality is not a typical European compromise, in which strict rules are adopted (to please supporters) but which are never enforced (to please opponents), because the political courage among ‘their colleagues’ to use them is lacking.

## Footnotes

1 Communication from the Commission to the European Parliament and the Council ‘A New EU Framework to Strengthen the Rule of Law’, COM(2014) 158 final.

2 Article 2 TEU.

3 Communication from the Commission to the European Parliament, the European Council and the Council ‘A new, modern Multiannual Financial Framework for a European Union that delivers efficiently on its priorities post-2020′, COM(2018) 98 final.

4 Matsuura (2015); Talbulescu and Goyeau (2013), p. 153.

5 The 2021–2027 MFF will be €1.074 trillion and will be accompanied by a €750 billion EU recovery fund, under the groundbreaking plan that will also see countries jointly borrow on the financial markets for the first time.

6 Kelemen and Scheppele (2018a, b). The situations of Poland and Hungary differ, since there are no well-proven cases that breaches of the rule of law by the Polish authorities have led to the misspending of EU funds (von Brauneck 2019, pp 37–60), while such cases have appeared in Hungary (Kelemen and Scheppele, op. cit.) and Romania (Pech and Kochenov 2019).

7 Scheppele and Pech (2018).

8 The case against Hungary (see C-286/12 *Commission v. Hungary*) concerned equal treatment of judges and prosecutors in employment relations. Cases against Poland (see C-619/18 *Commission v. Poland*;, C-192/18 *Commission v. Poland*; and C-791/19 *Commission v. Poland*, the latter case being still pending) have also concerned the independence of judges and the system of organization of the judiciary.

9 Case C-64/16 *Associacao Sindical dos Juizes Portugueses,* ECLI:EU:C:2018:117, para 32.

10 Uitz (2020, p. 8).

11 Schrank (2020).

12 2020 Rule of Law Report-Communication and country chapters, https://ec.europa.eu/info/publications/2020-rule-law-report-communication-and-country-chapters_en.

13 Uitz (2020, p. 7).

14 Closa and Kochenov (2016), Jakab and Kochenov (2017), Hatje (2018), Konstadinides (2017), Scheppele (2016), Šelih et al. (2017).

15 Uitz (2020, p. 4).

16 Goldner Lang (2020, p. 7).

17 Viă (2018).

18 Viţă (2017, p. 136).

19 Bartels (2005).

20 Council Implementing Decision 2012/156/EU of 13.03.2012 suspending commitments from the Cohesion Fund for Hungary with effect from 1 January 2013 (OJ L 78, 17.3.2012, p. 19).

21 Viţă (2018).

22 Uitz (2020, p. 16).

23 Cremona (2005, p. 19).

24 Article 1 Regulation no 2020/2092.

25 Recital 13 preamble and Article 3 of Regulation 2020/2092.

26 See July Conclusions (n 4) para A24, and annex to the conclusions, paras 22–23.

27 See the joint declaration by the Prime Ministers of Hungary and Poland, 26 November 2020, https://visegradpost.com/en/2020/11/26/eu-rule-of-law-joint-declaration-of-the-prime-minister-of-poland-and-the-prime-minister-of-hungary/

28 Tridimas (2020, p. XVI).

29 Regulation (EU, Euratom) 2018/1046 of the European Parliament and of the Council of 18 July 2018 on the financial rules applicable to the general budget of the Union, amending Regulations (EU) No 1296/2013, (EU) No 1301/2013, (EU) No 1303/2013, (EU) No 1304/2013, (EU) No 1309/2013, (EU) No 1316/2013, (EU) No 223/2014, (EU) No 283/2014, and Decision No 541/2014/EU and repealing Regulation (EU, Euratom) No 966/2012 (OJ L 193, 30.7.2018, p. 1).

30 Article 1 Financial Regulation 2018/1046.

31 Article 317 TFEU.

32 Article 2 (7) Financial Regulation 2018/1046.

33 Council Regulation (EU) 2020/2094 of 14 December 2020 establishing a European Union Recovery Instrument to support the recovery in the aftermath of the COVID-19 crisis (OJ L 433 l, 22.12.2020, p. 23).

34 To fund the Next Generation EU, the Commission will be able to borrow up to €750 billion in 2018 prices on the financial markets to be used for loans up to €360 billion and grants up to €390 billion. The capital raised is to be repaid by 2058. Article 5 of the Council Decision (EU, Euratom) 2020/2053 of 14 December 2020 on the system of own resources of the European Union and repealing Decision 2014/335/EU (OJ L 424, p. 1.).

35 Recital 7 preamble of Regulation 2020/2092.

36 Article 287 (2) 2, Article 310 (5) and Article 317 TFEU.

37 Article 310 (5) and Article 325 TFEU.

38 Article 317 TFEU.

39 Article 2 (59), Article 6 and Article 33 Financial Regulation 2018/1046.

40 Article 4 (3) TEU.

41 CJEU, cases C-138/03, C-324/03 and C-431/03 *Italy v. Commission*, ECLI:EU:C:2005:714, para 44; CFI, T-345/03 *Evropaïki Dynamiki v. Commission*, ECLI:EU:T:2015:168, para 77; CFI, T-549/08 *Luxemburg v. Commission*, ECLI:EU:T:2010:244, para 47; T-265/08 *Germany v. Commission*, ECLI:EU:T:2012:434, para 40; C-500/99 *P Conserve Italia v. Commission*, ECLI:EU:C:2002:45, para 88; T-308/05*, Italy v. Commission,* ECLI:EU:T:2007:382, para 109.

42 Recital 11 preamble of Regulation no 2020/2092.

43 Article 310 (5) and Article 325 TFEU.

44 Article (1) (a) Directive 2017/1371.

45 Recital 9 preamble of Regulation no 2020/2092.

46 Article 2 (a) Regulation no 2020/2092.

47 Recital 6 preamble of Regulation no 2020/2092.

48 Article 3 Regulation 2020/2092.

49 Article 4 (2) Regulation 2020/2092.

50 Recital 12 preamble of Regulation 2020/2092.

51 Recital 10 preamble of Regulation 2020/2092. See also recitals 3 and 9 preamble Regulation 2020/2092.

52 See footnote no 1.

53 Recital 15 of Regulation no 2020/2092.

54 von Bogdandy and Ioannidis (2014).

55 I. Golder Lang disagrees and claims that the dominant target of the Draft Regulation is not the protection of the EU budget, but the protection of the rule of law itself. Goldner Lang (2020, p. 11).

56 Article 317 TFEU.

57 Article 17 (1) TEU.

58 Actions necessary to implement the Union budget, such as e.g. selecting contractors, awarding grants, transferring Union funds, monitoring activities, etc. may be performed by the Commission directly (direct management) or by its agencies (indirect management). Article 62 (1) (a) and (c) Financial Regulation 2018/104.

59 Article 62 (1) (b) Financial Regulation 2018/104.

60 ‘Government entity’ means a public authority at any level of government, including national, regional and local authorities, as well as Member State organisations within the meaning of Article 2 (42) Financial Regulation.

61 Article 131(3) of the Financial Regulation.

62 Article 5 (a) Regulation 2020/2092.

63 Article 5 (b) Regulation No 2020/2092.

64 Article 5 (1) Regulation No 2020/2092.

65 COM(2018) 98 final.

66 The purpose of financial corrections is to protect the EU budget from the burden of erroneous or irregular expenditure. For expenditure under shared management, the task of recovering incorrectly made payments is primarily the responsibility of the Member State. Financial corrections can be implemented by the Member State through deducting irregular expenditure from the Member State’s payment claim, by the payment of a recovery order issued by the Commission, or by decommitment. The deduction can take two forms: withdrawal or recovery from beneficiaries.

67 Recital 17 preamble and Articles 5 (2) and 6 of Regulation 2020/2092.

68 COM(2018)393 final and COM(2018)375 final.

69 Article 4 (1) Regulation 2020/2092.

70 Article 5 (3) and Article 6 (7) and (7) Regulation 2020/2092.

71 Article 6 (6) Regulation 2020/2092.

72 Tiret 18 preamble and Article 5 (3) Regulation 2020/2092.

73 Conclusions of the European Council meeting, 10 and 11 December 2020, para I (2) (c).

74 Some state that the European Council had no legal base to require the Commission to adopt guidelines (not foreseen in the Regulation 2020/2021) nor to condition the application of this Regulation until the finalization of such guidelines. By doing so the European Council de facto intended to amend a legislative act, the adoption of which it has no role to play. Alemanno, Chamon, 2020.

75 Recital 26 preamble of Regulation 2020/2092.

76 See recital 16 preamble of Regulation 2020/2092.

77 The panel would be composed of specialists in constitutional law and financial and budgetary matters. Each national parliament would nominate one expert to this Panel and the European Parliament would appoint five experts. Representatives of relevant organisations and networks could be invited to the Panel as observers (Article 3a Draft Regulation, EP proposal).

78 Article 6 (1), (3) and (4) Regulation 2020/2092.

79 Article 6 (5), (6) and (7) Regulation 2020/2092.

80 Article 6 (9) Regulation 2020/2092.

81 Recital 27 and 28 preamble, Article 6 (1) and (2) and Article 8 Regulation 2020/2092.

82 Articles 289 and 294 TFUE.

83 Article 6 (11) Regulation 2020/2092.

84 The Council acts by QMV except where the Treaties provide otherwise (Article 16 (3) TEU).

85 Article 5 (6–8) draft Regulation (European Commission proposal).

86 Recital 15 preamble Draft Regulation. See critics: the ECA opinion No 1/2018, para 12.

87 Article 126 TFEU/

88 Under the SGP (‘preventive arm’), Member States submit yearly compliance reports that present expected fiscal development plans. If a Member State does not comply with the EU fiscal rules, an ‘Excessive Deficit Procedure’ (EDP) is initiated under the SGP (‘corrective arm’). If, despite multiple warnings, this situation is not corrected, sanctions can be imposed on this Member State.

89 Articles 4–6 Regulation 1173/2011, Article 3 Regulation 1174/2011, Article 10 (4) Regulation 1176/2011; Article 7 Fiscal Compact.

90 Article 7 (1) Regulation 2020/2092.

91 Recital 21 preamble of Regulation 2020/2092.

92 Article 7 (2) Regulation 2020/2092.

93 Recital 25 preamble and Article 7 (2) of Regulation 2020/2092.

94 Article 6 (5) Regulation 2020/2092.

95 Article 6 (6) Regulation 2020/2092.

96 Recital 22 preamble of the Regulation 2020/2092.

97 Article 6 (7) Regulation 2020/2092.

98 Article 6 (10) Regulation 2020/2092 and Article 237 TFEU.

99 Council Decision 2009/937/EU of 1 December 2009 adopting the Council’s Rules of Procedure (OJ L 325, 11.12.2009, p. 35).

100 Recital 23 preamble of Regulation 2020/2092.

101 Article 5 (1) (b) (i) Regulation 2020/2092.

102 Article 5 (1) (b) (ii) Regulation 2020/2092.

103 https://ec.europa.eu/info/sites/info/files/about_the_european_commission/eu_budget/eu-budget-glossary-2019_en.pdf

104 Article 7 (3) of Regulation 2020/2092 and Article 6 (3) of Council Regulation (EU, Euratom) 2020/2093 of 17 December 2020 laying down the multiannual financial framework for the years 2021 to 2027 (OJ L 433 l, 22.12.2020, p. 11).

105 Para 8 of the ECA opinion No 1/2018.

106 Article 5 (a) (i) Regulation 2020/2092.

107 Article 5 (a) (ii) Regulation 2020/2092.

108 Article 5 (a) (iv) Regulation 2020/2092.

109 Article 5 (a) (v) Regulation 2020/2092.

110 Article 5 (b) (iii) Regulation 2020/2092.

111 For criticism, see: the ECA opinion No 1/2018, para 27.

112 Recital 14 preamble and Article 4 (3) (a) and (b) of Draft Regulation (the European Parliament proposal).

113 COM(2018) 218 final.

114 Already now the Commission directly manages some EU funds used in external relations, e.g. the Union Action within Asylum, Migration and Integration Funds, Development Cooperation Instrument and the European Neighbourhood Instrument.

115 Article 5 (4) of the Regulation no 2020/2092.

116 Article 63 (b), Article 68 (1) and Article 98 of Proposal for a Regulation of the European Parliament and of the Council laying down common provisions on the European Regional Development Fund, the European Social Fund Plus, the Cohesion Fund, and the European Maritime and Fisheries Fund and financial rules for those and for the Asylum and Migration Fund, the Internal Security Fund and the Border Management and Visa Instrument, COM(2018) 375 final.

117 Recital 19 of preamble and Article 5 (2) of the Regulation 2020/2092.

118 Butler (2018, pp. 9–13), Pech and Kochenov (2019), Scheppele (2016).

119 Article 236 (3) (4) Financial Regulation 2018/1046.

120 Articles 23–24 Regulation 1303/2013.

121 Article 58 (2) Regulation 1306/2013.

122 Articles 72–74 and 122 (1) CP Regulation and Article 58 (2) 2 and Article 67–73 Regulation 1306/2013.

123 Article 83 CP Regulation and Article 90 draft CP Regulation.

124 Article 142 (a) CP Regulation and Article 91 draft CP Regulation.

125 Article 144 CP Regulation and Article 98 of draft CP Regulation.

126 Pech and Kochenov (2019*,* p. 11), Šelih et al. (2017, p. 7, 11).

127 The European Public Prosecution Office is an independent and decentralized prosecution office of the EU, with the competence to investigate, prosecute and bring to judgment crimes against the EU budget, such as fraud, corruption or serious cross-border VAT fraud. At this stage, there are 22 participating EU countries: Austria, Belgium, Bulgaria, Croatia, Cyprus, Czech Republic, Estonia, Finland, France, Germany, Greece, Italy, Latvia, Lithuania, Luxembourg, Malta, Netherlands, Portugal, Romania, Slovakia, Spain and Slovenia. Sweden has expressed its intent to join the EPPO. Council Regulation (EU) 2017/1939 of 12 October 2017 implementing enhanced cooperation on the establishment of the European Public Prosecutor’s Office (‘the EPPO’) (OJ L 283, 31.10.2017, p. 1).

128 Article 91(1)(d) of draft CP Regulation.

129 Schrank (2020).
